# Effects of Co-Fermentation with *Lactobacillus* and Yeast on the Structural and Physicochemical Properties of Millet Starch

**DOI:** 10.3390/foods15071186

**Published:** 2026-04-01

**Authors:** Xiaomin Guo, Muyao Lin, Jiaqi Liu, Haobo Chu, Mokhele Matsomoli Roslina, Nan Zheng, Xun Li, Yu Wang, Bo Nan, Xiujuan Wang, Chunhong Piao, Yuhua Wang, Xia Li

**Affiliations:** 1 College of Food Science and Engineering, Jilin Agricultural University, Changchun 130118, China; guoxiaomin@mails.jlau.edu.cn (X.G.); huanyun19770813@163.com (M.L.); 13614433389@163.com (J.L.); 15563142837@163.com (H.C.); matsomolimokhele@outlook.com (M.M.R.); littleseokjin@163.com (N.Z.); lixuncv@163.com (X.L.); wangyu526@jlau.edu.cn (Y.W.); nanbo@jlau.edu.cn (B.N.); juan4420@163.com (X.W.); yuhua-ww@163.com (Y.W.); 2 Jilin Province Innovation Center for Food Biological Manufacture, Jilin Agricultural University, Changchun 130118, China; 3 School of Food and Pharmaceutical Engineering, Guangxi Liupao Tea Modern Industry College, Wuzhou University, Wuzhou 543002, China; piaochunhong9111@163.com

**Keywords:** *Lactobacillus*, yeast, co-fermentation, millet starch, starch structure, physicochemical properties

## Abstract

Based on the previously screened high-performance strain *Lactobacillus* LP707, this study systematically investigated the effects of its co-fermentation with yeast on properties of millet starch. By comparing starch samples from unfermented, yeast-only fermented, *Lactobacillus*-*only* fermented and co-fermented treatments, it was found that co-fermentation reduced the amylose content of millet starch to 17.45% and shifted the molecular weight distribution toward lower values. Scanning electron microscopy revealed more pronounced surface erosion features on the co-fermented starch granules. X-ray diffraction and Fourier-transform infrared spectroscopy confirmed that co-fermentation did not alter the A-type crystalline pattern of starch; however, the short-range ordered structure ratio (1.45), relative crystallinity (20.78%), and gelatinization enthalpy (7.32 J/g) were significantly reduced, indicating dissociation of ordered structures. Pasting property analysis showed that the final viscosity and setback value of co-fermented starch decreased significantly. Low-field nuclear magnetic resonance analysis of water distribution indicated an increased proportion of free water with reduced mobility in the co-fermented starch gel. In vitro digestion confirmed higher hydrolysis rates and increased rapidly digestible starch content in co-fermented starch. In summary, co-fermentation with *Lactobacillus* LP707 and yeast more effectively modified properties of millet starch, providing a theoretical foundation for targeted functional improvement through microbial co-fermentation technology.

## 1. Introduction

Millet, a gluten-free grain product obtained from dehulled foxtail millet, belongs to the Poaceae family. It exhibits strong adaptability and can thrive in extreme weather conditions and poor soil environments [1]. Millet is not only rich in proteins, carbohydrates, minerals, and vitamins, but also contains trace elements such as iron, magnesium, and phosphorus. Furthermore, millet contains various bioactive compounds, including dietary fiber, polyphenols, flavonoids, and carotenoids [2]. Therefore, millet serves as an ideal healthy food ingredient, offering various health benefits such as improving spleen and stomach function, reducing blood lipids and pressure, protecting the liver, providing antioxidant and antitumor effects, and enhancing immune response [3].

Starch, a natural polysaccharide accounting for approximately 70% of the total weight of millet [4], is composed of amylose and amylopectin and serves as an important source of carbohydrates in the human diet [5]. The multiscale structure of starch modulates its gelatinization, gelation, and digestive properties by influencing water penetration, hydrogen bond formation and disruption, and the stability of ordered structures. For millet-based products, this structure–function relationship critically determines the thermomechanical resistance, hardness, viscoelasticity during processing, as well as the final texture and quality of the product, which serves as a fundamental theoretical basis for optimizing processing techniques and enhancing product quality.

Currently, the main methods for starch modification include physical modification (e.g., heat-moisture, high-pressure treatment [6] and ultrasound [7]), chemical modification (e.g., esterification or alkaline-alcohol treatment) [8], enzymatic modification [9], and combined modification (which involves two or more treatment methods). These modification approaches may have certain disadvantages in terms of processing complexity, cost, and environmental impact.

Over recent decades, numerous studies have demonstrated that fermentation can effectively modify the physicochemical and functional properties of starch. Fermentation is a common food processing technique widely used in the production of various daily consumed foods such as steamed buns, yogurt, soy sauce, and kimchi. Numerous studies have reported on the effects of fermentation on starch properties. For example, Ye et al. [10] found that spontaneous fermentation significantly reduced the amylose content and average molecular weight of sweet potato starch, resulting in a distinct branch chain length distribution pattern compared to native starch. Zheng et al. [11] reported that fermentation reduces the proportion of long chains in starch, thereby improving the gel texture properties of pea starch. Gu et al. [12] demonstrated that microbial fermentation systematically influences the multiscale structure, physicochemical properties, and functional performance of quinoa starch. Together, these findings indicate that fermentation, as a biological modification method, can effectively regulate the structure and properties of cereal starches, offering a potential approach for improving the quality of starch-based foods.

*Lactic acid bacteria* (LAB) and yeast are the most frequently utilized microorganisms in fermentation technology. LAB are widely distributed in nature and extensively utilized as probiotics. They play significant roles in enhancing food flavor, regulating gut microbiota, and boosting immune function [13]. Yeast plays a crucial role in the baking industry, serving as a key ingredient in the production of bread, cakes, and other flour-based products. It generates abundant aromatic compounds and imparts a soft, porous texture to the final products [14]. In previous studies, the fermentation-based modification of cereal starches has primarily focused on single microbial systems, such as the individual action of lactic acid bacteria or yeast, while systematic research on the effects of multi-strain co-fermentation on their physicochemical properties remains insufficient. Particularly in millet starch—a substrate of significant nutritional and processing value—the synergistic effects between *Lactobacillus* and yeast, as well as their regulatory mechanisms on the multiscale structure and functional properties of starch, have not yet been fully elucidated.

In our previous research, our laboratory isolated and screened a strain of *Lactobacillus* LP707 with excellent fermentation properties from traditionally spontaneously fermented millet dough. Further studies revealed that co-fermentation of this strain with yeast significantly improved the textural properties of millet steamed bread (Table S1) and enhanced its digestibility (Figure S1, Table S2). Based on these findings, this study systematically investigates the effects of co-fermentation with *Lactobacillus* LP707 and yeast on the molecular weight, granule morphology, crystalline structure, pasting properties, and digestibility of millet starch. The aim is to elucidate the synergistic mechanisms of multi-strain systems in starch modification, thereby contributing to the better utilization of millet grain resources and providing novel theoretical and technical foundations for developing functional millet starch and related products.

## 2. Materials and Methods

### 2.1. Preparation of Millet Starch Samples

#### 2.1.1. Materials

The millet flour used in this study was sourced from Mudanjiang City, Heilongjiang Province, China, and purchased from Heilongjiang Beichun Agricultural Product Development Co., Ltd., Mudanjiang, China.. The flour was sieved through an 80-mesh screen to ensure particle uniformity, and the fraction passing through the sieve was collected for further use. The *Lactobacillus* LP707 was preserved at the College of Food Science and Engineering, Jilin Agricultural University. The yeast used was Angel high-active dry yeast, produced by Angel Yeast Co., Ltd., Yichang, China. The strains were preserved in a medium containing glycerol and MRS broth, and stored at −80 °C. Biochemical reagents were purchased from Beijing Aoboxing Biotechnology Co., Ltd. (Beijing, China).

#### 2.1.2. Activation of *Lactobacillus* LP707 and Preparation of Bacterial Suspension

*Lactobacillus* LP707 was inoculated at 2% (*v*/*v*) into MRS broth and cultured under anaerobic conditions at 37 °C for 24 h. The strain was activated through three consecutive subcultures to ensure robust growth activity [15]. The activated bacterial culture was centrifuged at 4 °C and 4000 rpm for 10 min. The pellet was washed three times with sterile saline, followed by another centrifugation step to remove the supernatant [16]. The precipitated bacteria were then suspended in the same solution to obtain a bacterial suspension.

#### 2.1.3. Preparation of Fermented Millet Dough

A non-fermented millet dough sample was prepared by mixing millet flour with 70% (*v*/*w*) water and set aside. Yeast was inoculated at 1% (*w*/*w*) into the millet flour, mixed with 70% (*v*/*w*) water, and fermented in a constant temperature and humidity incubator at 30 °C for 2 h to obtain the yeast-fermented millet dough sample. A suspension of *Lactobacillus* LP707 was inoculated at 10% (*v*/*w*) into the millet flour, mixed with 60% (*v*/*w*) water, and fermented under the same conditions at 37 °C for 6 h to obtain the *Lactobacillus*-fermented millet dough sample. To prepare the co-fermented millet dough sample, the suspension was first inoculated at 10% (*v*/*w*) into the flour, mixed with 60% (*v*/*w*) water, and fermented at 37 °C for 6 h. Then, 1% (*w*/*w*) yeast was added to the fermented dough, mixed thoroughly, and fermentation was continued at 30 °C for an additional 2 h.

#### 2.1.4. Extraction of Millet Starch

Millet starch was extracted using a water-washing method according to a previously reported procedure with minor modifications [17]. The prepared millet dough samples were washed with distilled water and filtered through a 100 μm nylon sieve. The resulting slurry was allowed to settle, and the supernatant was discarded. The sediment was centrifuged at 6000 rpm for 10 min. After centrifugation, the supernatant was discarded, and the top yellowish layer was carefully removed with a spatula. This process was repeated at least three times until no yellow material remained. The white substance at the bottom was collected as starch. The purified starch was then dried in an oven at 37 °C for 24 h to obtain the millet starch samples.

The starch samples were designated as follows: UN (unfermented starch), YF (yeast-fermented starch), LF (*Lactobacillus* LP707 fermented starch), and CF (co-fermented starch).

### 2.2. Apparent Amylose Content (AAC)

The AAC values were determined using a visible spectrophotometric method according to the manufacturer’s instructions provided in the Amylose Content Assay Kit (BC 4260, Solarbio Science and Technology Co., Ltd., Beijing, China). A standard curve was prepared using amylose standards of known concentrations (provided in the kit), and the AAC values in the samples were calculated accordingly.

### 2.3. Chain Length Distribution in Amylopectin

Approximately 10 mg of millet starch sample was weighed and resuspended in 5 mL of water. The suspension was heated in a boiling water bath for 60 min with intermittent vortex mixing. Then, 50 μL of sodium acetate buffer (0.6 M, pH 4.4), 10 μL of NaN_3_ solution (2% *w*/*v*), and 10 μL of isoamylase (1400 U) were added, followed by incubation at 37 °C for 24 h. After enzymatic digestion, 0.5% (*w*/*v*) sodium borohydride solution was added, mixed by vortexing, and allowed to stand for 20 h. A 600 μL aliquot was transferred to a centrifuge tube and dried under a nitrogen stream at room temperature. The residue was dissolved in 30 μL of 1 M NaOH for 60 min, then diluted with 570 μL of water. The mixture was centrifuged at 12,000 rpm for 5 min, and the supernatant was collected.

The chain length distribution was analyzed using an ion chromatography system (ICS5000+, Thermo Fisher Scientific, Waltham, MA, USA) equipped with an electrochemical detector. Separation was performed on a Dionex™ CarboPac™ PA200 analytical column (250 × 4.0 mm, 10 μm) with an injection volume of 5 μL. The mobile phase consisted of A: 0.2 M NaOH and B: 0.2 M NaOH/0.2 M NaAC. The column temperature was maintained at 30 °C, and the flow rate was 0.4 mL/min. The elution gradient was as follows: 0 min, A/B (90:10, *v*/*v*); 10 min, A/B (90:10, *v*/*v*); 30 min, A/B (40:60, *v*/*v*); 50 min, A/B (40:60, *v*/*v*); 50.1 min, A/B (90:10, *v*/*v*); 60 min, A/B (90:10, *v*/*v*). Detection was carried out using an electrochemical detector.

### 2.4. Molecular Weight Determination

The molecular weight determination was performed with slight modifications based on the method described by Olennikov et al. [18]. Five milligrams of millet starch sample was dissolved in 5 mL of the mobile phase (DMSO) by heating at 80 °C for 3 h. The molecular weight was determined using a gel permeation chromatography system coupled with refractive index and multi-angle laser light scattering detectors (GPC-RI-MALS). A gel exclusion column with an appropriate molecular weight range was selected based on the properties of the compounds. The column temperature was maintained at 60 °C. The injection volume was 200 μL, and the mobile phase consisted of DMSO containing 0.5% LiBr. An isocratic elution was performed for 120 min at a flow rate of 0.3 mL/min. A dn/dc value of 0.07 mL/g was used for the DMSO solution.

### 2.5. Starch Color Measurement Analysis

The starch color measurement was performed with slight modifications based on the method described by Umebara et al. [19]. The color parameters of millet starch sample were measured using a colorimeter (CR-5, Konica Minolta, Tokyo, Japan). The instrument was calibrated with a white standard calibration plate prior to measurement. After calibration, each starch sample was placed on the sample stage and brought into close contact with the measuring probe. Color values were expressed in terms of L* (lightness), a* (red/green), and b* (yellow/blue). Each sample was placed at a random position, and the data were recorded to reflect the color variation in the starch.

### 2.6. Scanning Electron Microscopy (SEM)

All millet starch samples were dried in an oven at 45 °C for 12 h. An appropriate amount of each millet starch sample was mounted onto metal stubs using double-sided conductive adhesive tape and sputter-coated with gold under vacuum using a sputter coater (Emitech K550X, Quorum Technologies Ltd., Lewes, UK) [20]. The morphology of the dried starch was observed using a scanning electron microscope (TESCAN MIRA, Tescan Ltd., Shanghai, China). Imaging was performed at an accelerating voltage of 5.0 kV with magnifications of 1000×, 3000×, and 6000×. After identifying suitable and clearly visible starch granules, representative images with clear granular morphology were captured.

### 2.7. Particle Size Distribution Analysis

The particle size of millet starch samples was determined using a laser diffraction particle size analyzer (Mastersizer 2000E, Malvern Instruments Ltd., Malvern, Worcestershire, UK). The starch granules were suspended in distilled water and fully dispersed using an ultrasonic oscillator. The dispersion was then diluted with distilled water until an obscuration rate of 15~20% was achieved [21].

### 2.8. Fourier Transform Infrared Spectroscopy (FT-IR) Analysis

Analysis was performed using a Fourier transform infrared spectrometer (VERTEX 70, Bruker, Ettlingen, Germany) with slight modifications based on the method described by Correia et al. [22]. Briefly, 2 mg of millet starch sample was thoroughly mixed and finely ground with 200 mg of dried KBr powder, and pressed into a pellet using a mold. The instrument was calibrated with a pure KBr pellet as background. Spectra were collected over the wavenumber range of 600~4000 cm^−1^ at a resolution of 4 cm^−1^ with 24 accumulated scans. Spectral deconvolution was performed using OMNIC 8.0 software to improve resolution. The absorbance values at 1047 cm^−1^, 1022 cm^−1^ and 995 cm^−1^ were recorded, and their ratio was calculated.

### 2.9. X-Ray Diffraction (XRD) Analysis

The crystalline properties of millet starch were analyzed using an X-ray diffractometer (Rigaku SmartLab SE, Tokyo, Japan) operated at 40 kV and 40 mA. The scanning range was set from 5° to 40° (2θ) with a scanning rate of 3°/min [23]. All XRD patterns were processed and analyzed using Jade 6.0 software. The effect of fermentation on the crystalline structure of starch granules was evaluated, and the relative crystallinity (RC) was calculated according to the following formula [24]:

(1) $$RC=\frac{A(t)}{A(t)+B}$$

where *A*(*t*) represents the area of the crystalline region and *B* denotes the area of the amorphous region.

### 2.10. Differential Scanning Calorimetry (DSC) Analysis

The thermal properties of the millet starch samples were analyzed using a differential scanning calorimeter (DSC Q2000, TA Instruments, New Castle, DE, USA) according to the method described by Xiong et al. [25] with minor modifications. Exactly 5.0 mg of starch sample and 15 μL of distilled water were placed into an aluminum crucible, which was then hermetically sealed and equilibrated at 25 °C for 12 h. An empty crucible was used as a reference. The samples were heated from 25 °C to 120 °C at a rate of 10 °C/min. The onset temperature (To), peak temperature (Tp), conclusion temperature (Tc), and gelatinization enthalpy (ΔH) were recorded.

### 2.11. Pasting Property Analysis

The pasting properties of the starch samples were determined using a Rapid Visco Analyzer (RVA-4, Newport Scientific, Warriewood, NSW, Australia) according to the method described by Oyeyinka et al. [26] with minor modifications. Briefly, 3 g of millet starch sample was placed into an RVA canister, followed by the addition of 25 mL of distilled water. The slurry was mixed thoroughly to ensure homogeneity. The testing protocol consisted of the following steps: holding at 50 °C and 960 rpm for 1 min, heating to 95 °C at a rate of 12.16 °C/min and holding for 5 min, then cooling to 50 °C at the same rate and holding for an additional 2 min, followed by a final holding period at 50 °C for 7.5 min.

### 2.12. Texture Profile Analysis (TPA) Analysis

The gelatinized starch gel samples obtained after RVA testing were transferred into molds (30 mm in diameter, 30 mm in height), sealed with plastic film, and stored at 4 °C for 12 h. The refrigerated samples were then removed and allowed to equilibrate to room temperature. Each cylindrical sample was placed at the center of the sample platform, and TPA was performed using a texture analyzer (TA.new plus, ISENSO, Shanghai, China) equipped with a 36 mm aluminum cylindrical probe (Model P36R, 36 mm in both diameter and height). The test parameters were set as follows: a P/50 probe was used in TPA mode with a test speed of 1 mm/s and a trigger force of 5 g [27]. Each millet starch gel sample was measured by placing it at different positions.

### 2.13. Low-Field Nuclear Magnetic Resonance (LF-NMR) Analysis

Following the method described by Zhao et al. [28] with minor modifications, the gelatinized starch gel samples obtained from RVA analysis were cooled to room temperature, sealed with plastic film, and loaded into sample tubes. The water distribution was then determined using a low-field nuclear magnetic resonance analyzer (MESOMR23-040-V-1, Suzhou Niumag Corporation, Suzhou, China). The spin–spin relaxation time (T_2_) was measured using the Carr–Purcell–Meiboom–Gill (CPMG) pulse sequence. The main parameters were set as follows: number of scans (NS) is 8, waiting time (TW) is 6000 ms, number of echoes (NECH) is 18,000, and echo time (TE) is 0.3 ms. The relaxation decay data were inverted and analyzed using the instrument’s proprietary software.

### 2.14. In Vitro Digestibility Properties

Referring to the in vitro simulated digestion method reported by Gu et al. [12] with slight modifications, the detailed procedure was as follows: precisely 50 mg of millet starch was weighed into a 50 mL sterile centrifuge tube. After adding 2 mL of distilled water for uniform dispersion, 3 sterile glass beads and 1 mL of α-amylase solution (50 U/mL) were introduced. The mixture was pre-incubated in a constant-temperature water bath shaker at 37 °C and 120 rpm for 2 min. Then, 5 mL of pepsin solution (3500 U/mL) was added and digestion was carried out for 30 min. Subsequently, 5 mL of 0.02 M sodium hydroxide solution, 20 mL of sodium acetate buffer (pH 6.0), and 5 mL of pancreatin-amyloglucosidase mixture (containing 4 mg/mL pancreatin and 26 U/mL amyloglucosidase) were added sequentially.

At time points of 0, 10, 20, 30, 60, 90, 120, 180, 240, and 300 min, 1 mL of the digest was collected and immediately mixed with 1 mL of absolute ethanol to terminate enzyme activity. After centrifugation at 4000 rpm for 5 min, 1 mL of the supernatant was taken for determination using the DNS colorimetric method. A hydrolysis rate curve was plotted, and the contents of rapidly digestible starch (RDS), slowly digestible starch (SDS), and resistant starch (RS) were calculated according to the following formulas:

(2) Starch hydrolysis rate (%) = G × 0.9/WS × 100

(3) $$\mathrm{RDS}(\%)\;=\;G_{20}\;\times \;0.9/\mathrm{WS}\;\times \;100$$

(4) $$\mathrm{SDS}(\%)\;=\;\left(G_{120}-G_{20}\right)\;\times \;0.9/\mathrm{WS}\;\times \;100$$

(5) RS(%)=100 − (RDS+SDS)

where G is the amount of glucose released at the sampling time point; G_20_ is the glucose released within 20 min; G_120_ is the glucose released within 120 min; WS is the sample weight; and the hydrolysis index of the standard is defined as 100.

### 2.15. Data Analyses

All experiments in this study were performed in triplicate, and the experimental data are expressed as mean ± standard error. Statistical analysis was conducted using one-way analysis of variance (ANOVA) followed by Duncan’s multiple range test (*p* < 0.05) with SPSS 27.0 software to determine significant differences among means. Figures were generated using Origin 9.8 software.

## 3. Results and Discussion

### 3.1. AAC Analysis

As shown in Table 1, the AAC of starch extracted from all fermented millet dough samples was significantly lower than that UN sample (*p* < 0.05). A similar trend has been reported by Chang et al. [29]. The AAC of the UN sample was 24.07%, while that of the fermented samples ranged from 17.45% to 20.34%. Specifically, the AAC values were 20.34% for YF sample, 19.55% for LF sample, and 17.45% for CF sample, which exhibited the lowest AAC. The organic acids produced by lactic acid bacteria, particularly lactic acid, are capable of penetrating the amorphous regions of starch granules, disrupting hydrogen bonds, and promoting the hydrolysis of amylose into shorter, soluble fragments [30]. Additionally, enzymatic activities might further degrade the amorphous regions, contributing to the decreased amylose content [10]. The co-fermentation process resulted in a more pronounced reduction in AAC compared to individual fermentations with yeast or *Lactobacillus* alone. This synergistic effect may be attributed to the complementary metabolic activities of these two microbial species: lactic acid bacteria produce organic acids and enzymes that facilitate the breakdown of starch granules, while yeast consumes readily available sugars and generates metabolites that may further influence starch structure. The enhanced acidity and enzymatic diversity in the co-culture system likely promote more extensive disruption of the starch architecture, leading to greater degradation of amylose.

### 3.2. Starch Chain Length Distribution

The chain length distributions of millet starch under different fermentation conditions are presented in Table 2. Based on the degree of polymerization (DP) range, starch chains can be classified into four categories: A chains (DP 6–12), B_1_ chains (DP 13–24), B_2_ chains (DP 25–36), and B_3_ chains (DP ≥ 37). Data analysis indicated that fermentation methods significantly modulated the chain length distribution of millet starch. After co-fermentation, the proportion of A and B_1_ chains increased significantly, the proportions of B_2_ and B_3_ chains showed a declining trend (*p* < 0.05). Since amylose can be structurally regarded as an extremely long B_3_ chain, the reduction in B_3_ chain content may be attributed to the action of lactic acid and microbial enzymes produced by *Lactobacillus* during fermentation. As shown in Table 1, this process promoted the degradation of amylose, leading to its breakdown into shorter chain segments (such as A and B_1_ chains). This resulted in an increased proportion of these shorter chains in the fermented samples and supports the hypothesis that fermentation facilitates chain cleavage within both amorphous and crystalline regions. This finding aligns with previous research by Bian et al. [31]. They reported similar alterations in chain length distribution following microbial fermentation.

Meanwhile, the concurrent decrease in B_2_ chain content may also be attributed to their partial hydrolysis into shorter fragments, further contributing to the increased levels of A and B_1_ chains. These shifts in chain length distribution reflect a depolymerization process that preferentially targets longer chains, thereby reshaping the molecular structure of the starch. It should be noted that starch chain length distribution can be influenced not only by fermentation conditions but also by external factors such as light exposure during sample handling [32]. However, in this study, all samples were processed under controlled conditions to minimize the impact of such confounding variables.

The results of this study demonstrate that, compared to other fermentation methods, co-fermentation had the most pronounced effect on the chain length distribution of millet starch. These structural alterations are expected to significantly impact the physicochemical properties and subsequent processing performance of the starch.

### 3.3. Molecular Weight and Radius of Gyration

The molecular weight and radius of gyration of millet starch under different fermentation treatments are shown in Table 2. Compared with the UN sample, the weight-average molecular weight (Mw) of fermented starch decreased significantly (*p* < 0.05), indicating chain cleavage during fermentation and a shift in the overall molecular weight toward lower values, which directly confirms starch degradation [33]. Among the samples, the CF sample exhibited the lowest Mw value of 5.86 × 10^4^ g/mol. The number-average molecular weight (Mn) also reflects the extent of chain scission; a lower Mn indicates a higher proportion of small fragments. CF sample showed the lowest Mn value of 1.34 × 10^4^ g/mol, suggesting the most pronounced degradation under this condition. In contrast, the Mn of UN sample was 3.31 × 10^4^ g/mol, while that of YF sample slightly increased to 3.46 × 10^4^ g/mol. This increase may be attributed to the rapid consumption of small-molecular sugars by yeast, which reduces the “dilution” or “isolation” effect of soluble sugars on starch chains, thereby promoting local aggregation via hydrogen bonding and resulting in an apparent rise in molecular weight. These findings are consistent with previous studies reporting decreased Mw and Mn in rice starch fermented with lactic acid bacteria [34], supporting the general impact of microbial fermentation on cereal starch molecular weight. The breadth of molecular weight distribution can be described by the polydispersity index (Mw/Mn). A higher Mw/Mn value indicates a wider distribution and greater heterogeneity in molecular size [35]. As shown in Table 2, CF and LF samples displayed higher Mw/Mn ratios, implying non-uniform degradation during fermentation. This non-uniformity likely arises from structural heterogeneity of starch granules (e.g., uneven distribution of crystalline and amorphous regions), localized action of microbial enzymes, or diffusion limitations, leading to asynchronous chain cleavage at different sites [31]. Additionally, the decrease in radius of gyration (Rz) after fermentation (e.g., 161.75 nm for CF sample) aligns with chain breakage and conformational compaction, collectively reflecting reduced structural looseness of starch molecules. Alterations in molecular weight and its distribution directly influence starch physicochemical properties, such as gelatinization behavior, gel strength, and digestibility. Therefore, fermentation modifies the molecular structure of millet starch, which may subsequently affect its functional performance in food processing and the quality of the final product. This provides a theoretical basis for tailoring the processing adaptability of millet starch.

### 3.4. Analysis of Starch Color

Table 1 presents the color parameters (L*, a*, b* and ΔE) of different millet starch samples. It can be observed that all starch samples exhibited high L* values (>95), indicating high purity of the starch. The results indicate that compared to UN sample, all starch samples extracted from fermented millet dough showed increased L* and a* values, and decreased b* values. The significant increase in L* value suggests that certain impurities such as proteins, fats, and cellulose in the starch were degraded during fermentation, leading to improved brightness of the fermented products. Ilowefah et al. [36] observed that fermentation increased the brightness of brown rice flour. Similarly, Gong et al. [37] reported an increase in the L* value of fermented purple sweet potato powder, resulting in a lighter purple appearance. The b* values of starch from fermented millet dough decreased significantly (*p* < 0.05), indicating a reduction in yellowness. This result is consistent with the findings reported by Díaz et al. [38], who noted that natural fermentation reduced the b* value of starch compared to the original native sweet potato starch. Millet is naturally rich in yellow pigments such as carotenoids and lutein [39]. Under acidic conditions (low pH), the chromophore structures of these pigments may be altered, leading to a decrease in yellow saturation, which is macroscopically reflected as a reduction in b* value. Furthermore, notable differences in ΔE values were observed among the millet starch samples (*p* < 0.05). The highest ΔE value was recorded for CF sample, suggesting that co-fermentation had the most pronounced effect on the color parameters of millet starch.

### 3.5. Microstructure

**Figure 1:**
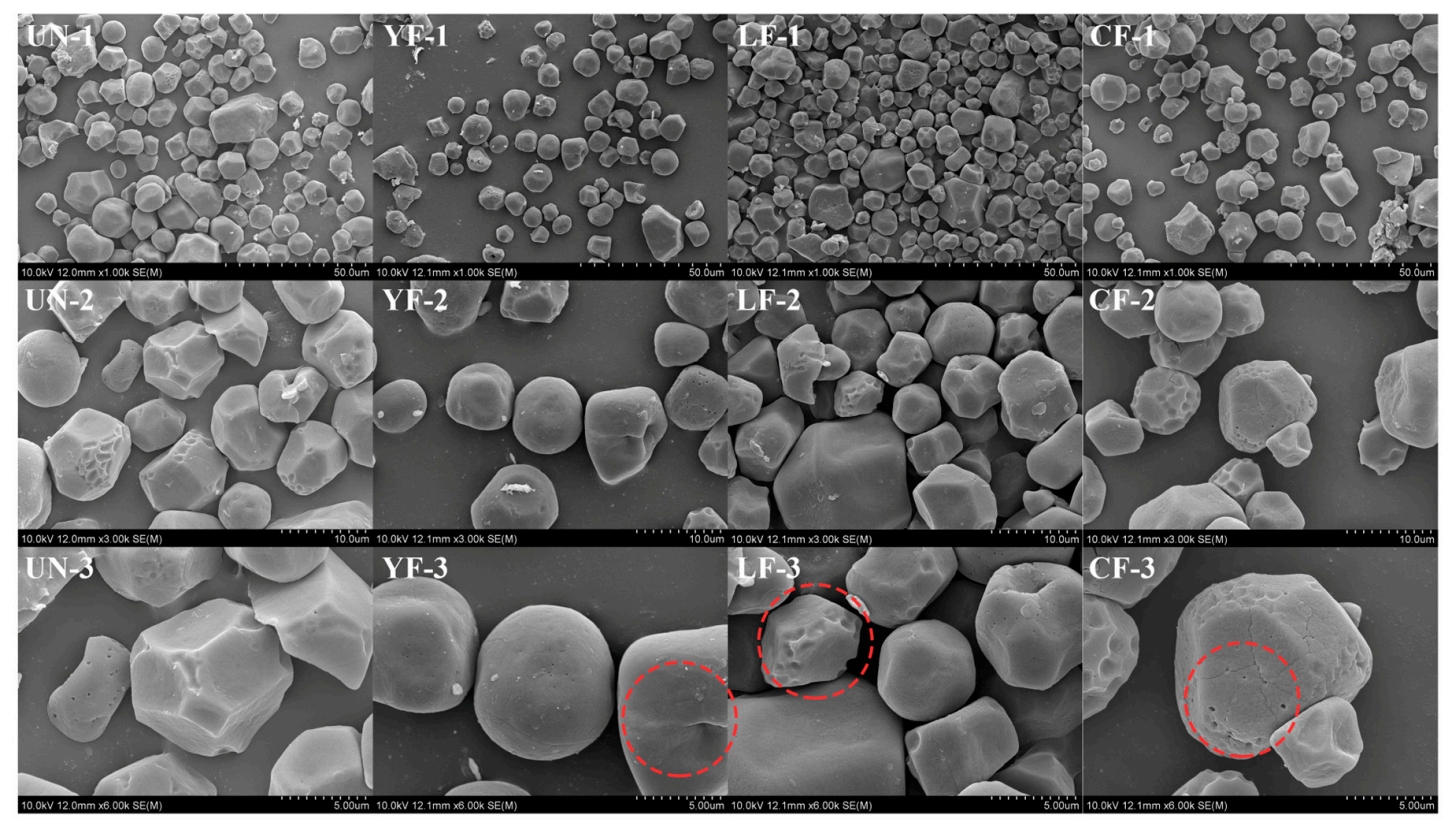
Microstructure of millet starch extracted using different fermentation methods: (1) 1000×; (2) 3000×; (3) 6000×. (The red circles in the figure highlight the representative microstructure of millet starch granules after fermentation).

Figure 1 presents the SEM images of millet starch granules subjected to different fermentation treatments. All samples exhibited typical round, polygonal, and irregularly shaped granules, which are characteristic morphological features of millet starch. However, notable differences in surface characteristics were observed among the treatment groups. Microbial fermentation clearly induced physical disruption on the surface of starch granules. Compared with the UN sample, which exhibited relatively smooth and intact surfaces, the fermented samples displayed increased surface pitting, cracking, and fragmentation. These morphological alterations were particularly pronounced in the CF sample, where granules showed extensive erosion. Similar phenomena have been reported by Xu et al. [40], who observed that raw potato starch granules had a smooth surface, whereas those fermented with Lactobacillus exhibited obvious erosion with small pores and depressions. Hamid et al. [41] also reported notable changes in the structural characteristics of pearl millet after 7 days of fermentation, including partial degradation of granules and visible physical indentations. These findings collectively support the view that fermentation actively alters the morphology of starch granules.

The observed surface disruption can be attributed to the synergistic action of multiple factors during the fermentation process. Previous studies have suggested that CO_2_ produced by yeast fermentation disrupts the starch network structure, while secreted glucoamylases directly act on the granules, creating a “honeycomb-like” erosion that leads to cracking or fragmentation. Meanwhile, fermentation by lactic acid bacteria reduces the pH, contributing to acid hydrolysis and enzymatic degradation that result in localized corrosion and pores on the granule surface [42]. This co-fermentation system likely amplifies these effects through the combined action of acid production, enzymatic activity, and physical disruption, resulting in the most pronounced granular morphological changes observed in the CF sample. Such structural modifications at the granule surface level are expected to influence the functional properties of starch, including gelatinization behavior and digestibility. Furthermore, a looser and more porous granular structure may contribute to improved textural characteristics in millet-based products such as steamed bread, bread, and fermented pastries, potentially enhancing product softness and overall sensory quality.

### 3.6. Particle Size Distribution

**Figure 2:**
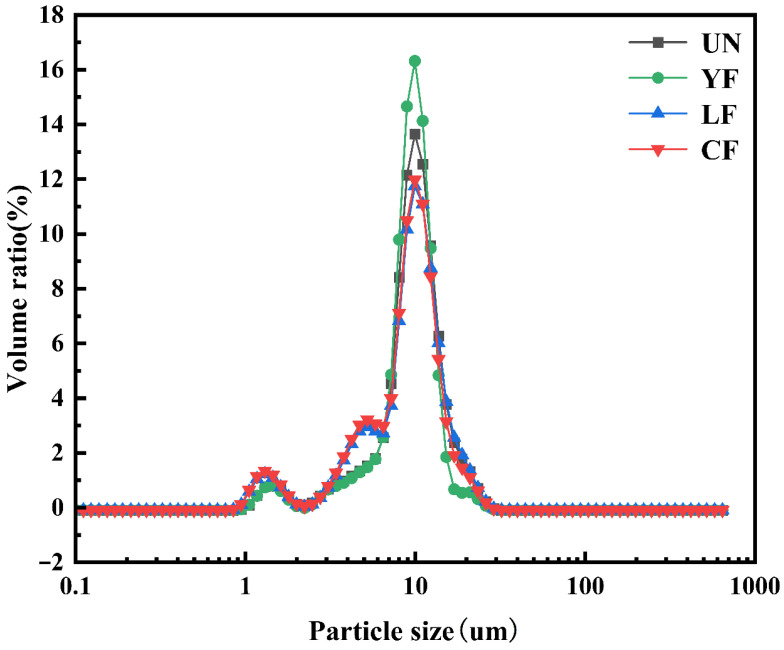
Particle size distribution of millet starch extracted using different fermentation methods.

Table 1 summarizes the changes in the particle size distribution of millet starch under different fermentation conditions, with the corresponding volume distribution curves shown in Figure 2. The parameters d_(0.1)_, d_(0.5)_, and d_(0.9)_ represent the particle diameters at 10%, 50%, and 90% of the cumulative volume distribution, respectively. Compared with the UN sample, the d_(0.5)_ values of all fermented samples decreased significantly (*p* < 0.05), indicating that fermentation reduced the overall particle size of starch granules. This phenomenon may be attributed to the action of microbial metabolites (e.g., organic acids and enzymes) on the surface and internal structure of starch granules during fermentation, leading to surface erosion, partial degradation, and particle fragmentation. This trend aligns with the reduction in d_(0.5)_ observed for highland barley starch after fermentation as reported by AL-Ansi et al. [43].

Further analysis of the particle size distribution characteristics shows that d_(0.1)_ reflects changes in the small particle population. The d_(0.1)_ values of LF and CF samples decreased significantly (*p* < 0.05), indicating the generation of more fine particles during fermentation, likely due to uneven surface degradation or localized structural disintegration leading to particle fragmentation. The parameter d_(0.9)_ reflects changes in the large particle population. The decrease in d_(0.9)_ for YF and CF samples suggests structural loosening and size reduction in some large granules during fermentation, while a slight increase in d_(0.9)_ for the LF sample may be related to particle swelling or local aggregation.

The breadth of particle size distribution can be described by the Span value. Higher Span values for CF and LF indicate a wider and more heterogeneous size distribution, pointing to non-uniform degradation of starch granules during fermentation. This trend is consistent with the increase in Mw/Mn ratios shown in Table 2, together confirming the heterogeneous structural changes induced by fermentation. In contrast, YF exhibited the lowest Span value, suggesting a more concentrated and uniform particle distribution.

The specific surface area (SpSA) reflects the theoretical surface area of the particles. The CF sample showed the highest SpSA value (307.80 m^2^/kg), indicating finer or more irregular particle surfaces, which increases the contact area with the surrounding environment and may affect hydration properties, enzymatic hydrolysis rates, and functional performance.

In summary, fermentation significantly altered the particle size and distribution characteristics of millet starch, which may influence the processing behavior and final quality of related products.

### 3.7. FTIR Analysis

**Figure 3:**
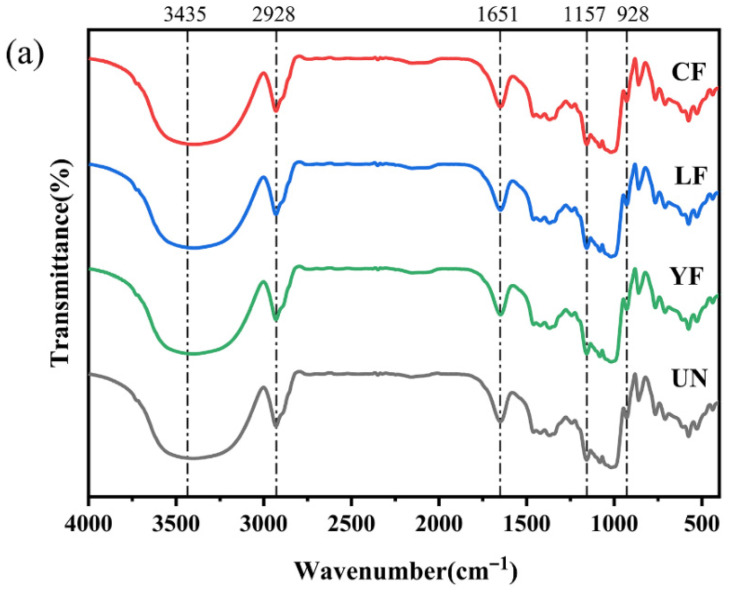

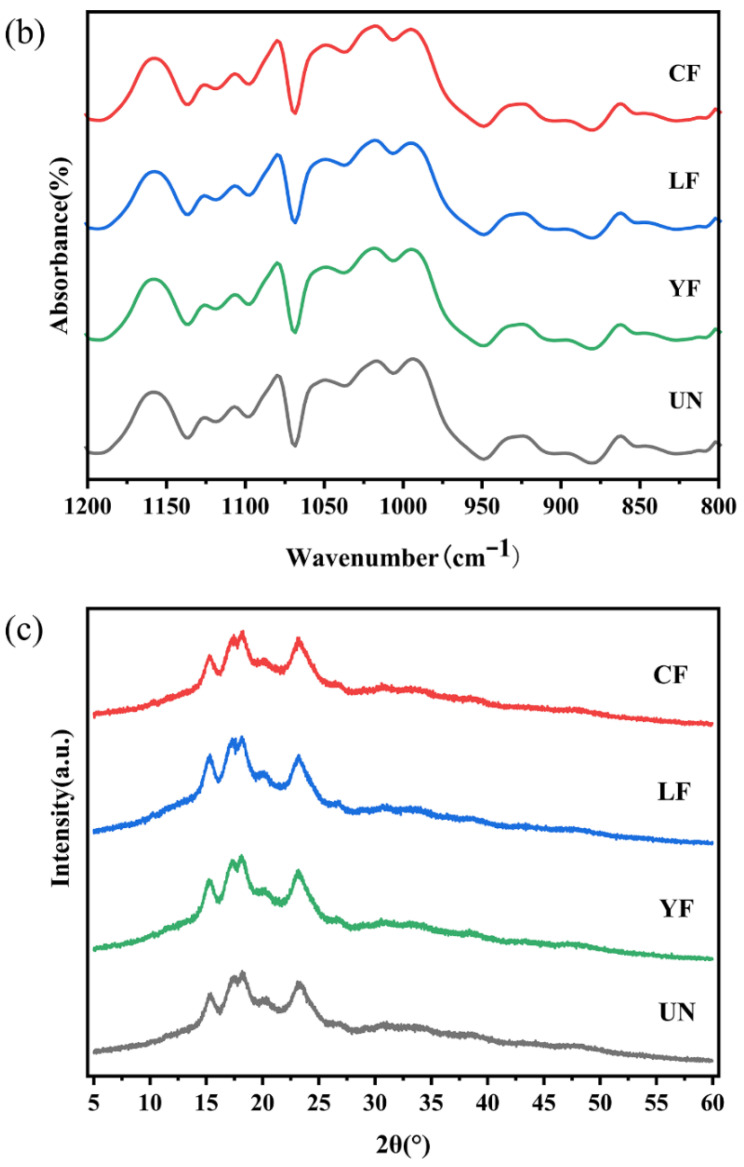
Structural properties of millet starch extracted using different fermentation methods. (**a**) FTIR spectra (4000–500 cm^−1^); (**b**) Deconvoluted FTIR spectra; (**c**) XRD patterns.

FTIR spectroscopy is commonly used to characterize the short-range ordered structure of starch. As shown in Figure 3a, all millet starch samples displayed similar characteristic absorption peaks and spectral profiles in the range of 4000–500 cm^−1^, with no new peaks appearing, indicating that fermentation did not alter the fundamental chemical structure of starch or introduce new functional groups.

Figure 3b presents the peak-fitting results of the FTIR spectra in the region 950–1100 cm^−1^. The band at 995 cm^−1^ is associated with intramolecular hydrogen bonds formed by the C6 hydroxyl groups of starch, reflecting the compactness of the helical structure. The band at 1022 cm^−1^ mainly corresponds to vibrational modes in the amorphous regions of starch [44], whereas the band at 1047 cm^−1^ is attributed to the characteristic absorption of ordered crystalline structures. Typically, the intensity ratio of 1047 cm^−1^ to 1022 cm^−1^ (R_1047_/_1022_) reflects the relative proportion of crystalline to amorphous regions in starch, while the ratio of 995 cm^−1^ to 1022 cm^−1^ (R_995_/_1022_) is used to describe the integrity and stability of the helical structure.

As shown in Table 1, after fermentation, the R_1047_/_1022_ values of millet starch generally decreased, indicating a relative reduction in crystalline regions and a corresponding increase in amorphous regions. Meanwhile, the decline in R_995_/_1022_ values suggests possible dissociation or reduced order of starch helical structures, further supporting the weakening of short-range ordered structure during fermentation [45].

In addition, typical starch FTIR spectra show a broad absorption band near 3435 cm^−1^ attributed to O-H stretching vibrations, a band near 2928 cm^−1^ corresponding to saturated C-H stretching, an absorption near 1651 cm^−1^ assigned to C=O vibrations of bound water or carboxyl groups, a band near 1157 cm^−1^ belonging to C-O-C stretching, and an absorption near 928 cm^−1^ related to the vibrational mode of α-1,4-glycosidic bonds [46]. These characteristic peaks were consistently present in all samples, confirming that fermentation did not significantly alter the basic molecular conformation of starch; its primary effect lies in the reorganization of higher-order structure and changes in the degree of order.

In summary, FTIR analysis indicates that fermentation mainly weakens the crystalline regions and helical ordered structure of starch while increasing the proportion of amorphous regions, thereby influencing its physicochemical properties and processing functionality. This provides structural level evidence for understanding the molecular mechanisms by which fermentation modifies starch properties.

### 3.8. Crystalline Structure

The XRD patterns of the millet starch samples are shown in Figure 3c. All samples exhibited distinct diffraction peaks at 2θ angles of approximately 15°, 17°, 18°, and 23°, which are characteristic of an A-type crystalline structure. This observation indicates that fermentation with yeast, lactic acid bacteria, or their combination did not alter the crystalline polymorphism of millet starch. These results are consistent with the findings of Tu et al. [47], who also reported no change in the crystalline structure of rice starch after fermentation. Although fermentation modified the granule morphology, particle size distribution, and amylopectin chain length profile of the starch, the XRD analysis revealed that the positions and assignments of the characteristic peaks remained unchanged. This suggests that fermentation primarily affected the amorphous regions of the starch granules, without disrupting the molecular arrangement in the crystalline domains. The relative crystallinity values calculated from the XRD patterns are summarized in Table 1: UN (26.44 ± 0.10%), YF (24.83 ± 0.08%), LF (22.25 ± 0.09%), and CF (20.78 ± 0.12%). The relative crystallinity decreased progressively with different fermentation treatments. This trend may be attributed to the disruption of hydrogen bonds within the starch, leading to the degradation of microcrystals, structural depolymerization, and a consequent reduction in granule integrity [48]. The combined metabolic activities of lactic acid bacteria and yeast during co-fermentation may have intensified these effects, leading to a significant reduction in crystallinity in the CF sample.

### 3.9. DSC Analysis

The thermal characteristic parameters of millet starch subjected to different fermentation treatments are listed in Table 3. The characteristic values of To, Tp, Tc and ΔH were determined by DSC in the range of 25–120 °C. These parameters collectively reflect the thermal stability and gelatinization behavior of starch, and serve as important determinants of its functional properties in food processing applications. Studies have shown that gelatinization temperature is primarily regulated by amylose content, branch structure, and the relative proportion of crystalline to amorphous regions in starch granules. Generally, starch with higher crystallinity and tightly arranged double helices exhibits a higher gelatinization temperature. This is because more thermal energy is required to disrupt its ordered structure.

Compared with the UN sample, the To values of all fermented starch samples decreased significantly (*p* < 0.05). The CF sample showed the lowest To (64.51 °C). The decrease in To indicates dissociation of the crystalline structure and reduced thermal stability of starch granules during fermentation, which may be related to molecular chain degradation, weakened short-range order (e.g., the decrease in the R_1047_/_1022_ ratio from FTIR analysis), and an increased proportion of amorphous regions induced by fermentation.

Meanwhile, Tp and Tc generally increased in the fermented samples. The rise in Tp suggests that higher energy input is required to completely melt the starch crystals during gelatinization, which may be attributed to the reorganization of starch chains into more stable locally ordered regions after fermentation. The increase in Tc reflects a broader temperature range of the gelatinization process, indicating more diffuse melting behavior and enhanced structural heterogeneity in fermented starch. This trend was particularly pronounced in the LF sample, which exhibited the highest Tp and Tc.

The ΔH value reflects the energy consumed during the disruption of double-helical structures upon melting of starch crystals [49]. The ΔH of all four millet starch samples decreased significantly (*p* < 0.05), indicating that fermentation reduced the overall content of ordered structures, including crystalline and double-helical regions. This result is consistent with the reported trend of decreasing ΔH in highland barley starch with extended fermentation time [43], further confirming the disordering and crystalline weakening induced by fermentation. The reduction in ΔH is not only directly related to decreased crystallinity (Table 1) but also associated with the dissociation of helical structures and possible chain reorganization and non-uniform degradation during fermentation.

In summary, fermentation significantly altered the thermal response characteristics of millet starch by lowering To and ΔH while raising Tp and Tc. Collectively, these changes indicate a structural transition from a compact, uniformly ordered state to a looser, more irregular and disordered morphology. These thermal alterations have significant implications for the processing behavior of fermented millet starch, potentially influencing its paste viscosity, gel properties, and ultimately the textural quality of millet-based food products.

### 3.10. Pasting Properties

The pasting properties of starch, as determined by a Rapid Visco Analyzer (RVA), are closely related to the sensory quality of starch-based foods. The pasting characteristics of the millet starch samples are summarized in Table 4. During heating, starch molecules undergo a transition from an ordered to a disordered state and become dispersed in water. When the starch suspension is heated to a specific temperature, the granules are progressively disrupted, absorb water, and swell, leading to a sharp increase in viscosity and the formation of a viscous starch paste [50].

Pasting Temperature (PT) reflects the thermal threshold at which starch granules begin to swell and rupture significantly. In this study, the PT values of all samples ranged from 77.25 to 81.60 °C, with LF and CF samples showing higher PT values of 81.60 °C and 79.88 °C, respectively. This indicates that fermentation enhanced the structural stability of starch granules, making them more resistant to disruption under heat and shear. This phenomenon may be related to surface densification, reorganization of internal crystalline regions, or changes in branch structure induced by fermentation [51].

Peak Viscosity (PV) corresponds to the maximum viscosity reached when starch granules are fully swollen. The LF sample exhibited the lowest PV value of 2567 cP, significantly lower than that of the UN sample of 3629 cP (*p* < 0.05), suggesting restricted swelling capacity and a more compact structure during gelatinization. This is likely associated with molecular chain degradation, enhanced ordered structure, and changes in granule surface morphology caused by fermentation, consistent with reports of decreased PV in fermented potato starch [40].

Trough Viscosity (TV) represents the minimum viscosity observed during cooling after reaching peak viscosity. The TV value of UN was 1836.67 cP. All fermented millet starch samples showed significant changes in trough viscosity (*p* < 0.05). Specifically, LF and CF samples displayed markedly lower TV values, with CF being the lowest at 1320.00 cP, while the YF sample showed a slight increase of 1910.00 cP. These differences reflect the distinct impacts of different fermentation methods on starch structure and pasting behavior.

Final Viscosity (FV) indicates the viscosity formed during cooling due to rearrangement of amylose and reorganization of amylopectin. Fermentation significantly reduced the FV values of the samples (*p* < 0.05), with CF sample showing the lowest FV value of 2544.33 cP. This suggests that co-fermentation effectively inhibited starch retrogradation, which may help maintain textural stability and delay staling during storage. This result aligns with previous findings of generally decreased FV in fermented millet starch [52].

Breakdown (BD) reflects the stability of starch paste under high temperature and shear, resistance to shear thinning. The BD values of all fermented samples decreased significantly, with LF showing the lowest BD value of 1065.67 cP, indicating that fermentation significantly enhanced the thermal stability of the starch paste and slowed down granule rupture and molecular chain dissociation. This trend is consistent with the decrease in BD of foxtail millet starch observed with extended fermentation time [29].

Setback (SB) reflects the tendency of starch paste to recrystallize after cooling, short-term retrogradation ability. The SB value of CF sample decreased significantly to 1224.33 cP, and all fermented samples exhibited lower SB values than UN sample, indicating that fermentation effectively inhibited starch rearrangement and gelation. Retrogradation is mainly influenced by amylose content and its structure. As shown in Table 1, the proportion of amylose decreased after fermentation, and its molecular structure was altered, collectively contributing to the reduction in SB. This is consistent with the reported decrease in SB of starch due to fermentation [53], further confirming that fermentation modulates retrogradation behavior by regulating amylose characteristics.

In summary, fermentation systematically altered the gelatinization and retrogradation behavior of millet starch by increasing PT, decreasing PV and BD, and modulating FV and SB. These changes collectively indicate that fermentation not only enhanced the structural stability of starch granules and the thermal stability of the paste but also effectively suppressed retrogradation tendency, thereby providing an important theoretical basis for improving the processing adaptability, textural quality, and storage performance of millet starch-based foods.

### 3.11. Gel Textural Properties

Table 5 presents the changes in textural properties of millet starch gels after aging at 4 °C following different fermentation treatments. No significant differences were observed in hardness or recoverability among all samples (*p* > 0.05), indicating that fermentation did not markedly alter the maximum resistance of the gels to external pressure or their recovery after deformation. However, compared with the UN sample, fermented millet starch gels showed significant decreases in springiness, chewiness, adhesiveness, and cohesiveness (*p* < 0.05), suggesting that fermentation distinctly affected the continuity and structural stability of the gel network.

These changes in textural characteristics are closely related to the structural modifications of starch induced by fermentation. On one hand, microbial metabolism during fermentation may cause local degradation of starch granules, resulting in porous or rough surface structures that weaken granule integrity [52], thereby reducing the structural coherence and cohesive strength of the gel during deformation. On the other hand, gases possibly generated during fermentation could form micro-bubbles within the gel matrix, disrupting the continuity of the gel phase and consequently lowering its springiness and chewiness.

Furthermore, the degradation of starch molecular chains and the weakening of short-range ordered structures after fermentation may also affect the rearrangement and cross-linking of starch molecules during gelatinization and aging, leading to a looser gel network that macroscopically manifests as reduced adhesiveness and cohesiveness.

It is noteworthy that the extent of influence varied among different fermentation methods. The LF sample exhibited the lowest values in springiness, chewiness, adhesiveness, and cohesiveness, indicating the most loosely structured gel. In contrast, although the CF sample also showed significant reductions in all these parameters compared to UN sample, its overall performance was better than that of LF sample. This may reflect a more complex interaction between yeast and lactic acid bacteria during co-fermentation, which establishes a certain balance between degradation and reorganization of starch structure.

### 3.12. LF-NMR Analysis

LF-NMR can be used to track water state and migration in starch gels during retrogradation. After gelatinization, starch undergoes retrogradation during cooling and storage [54], a process that directly affects the textural stability, shelf life, and sensory quality of starch-based foods. The transverse relaxation time (T_2_) reflects the mobility of water molecules in the system: a higher T_2_ value indicates greater freedom of water motion, whereas a lower T_2_ suggests stronger interactions between water and the matrix, resulting in restricted mobility [55]. Based on the relaxation time distribution, water in the system can typically be classified into three types: tightly bound water (T_21_, approximately 0.1–10 ms), weakly bound water (T_22_, approximately 10–100 ms), and free water (T_23_, generally >100 ms).

**Figure 4:**
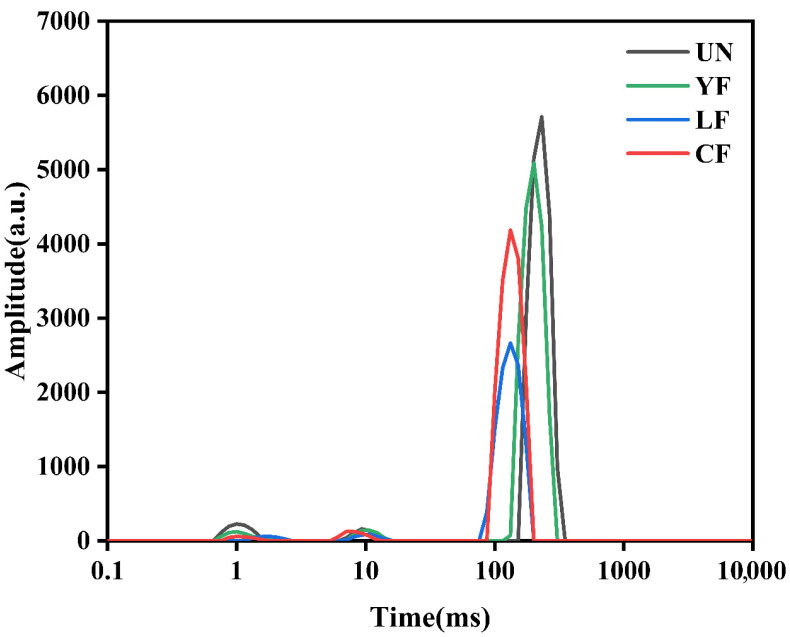
LF-NMR Relaxation time of millet starch gels extracted using different fermentation methods.

As shown in Table 6 and Figure 4, the T_23_ of all fermented millet starch gels shifted significantly to the left compared with the UN sample, indicating a shortened relaxation time and reduced mobility of free water. This change suggests that fermentation enhanced the interactions between starch molecules and water, allowing free water to be more effectively trapped within the gel network. The CF sample exhibited the lowest T_23_ of 132.19 ms and the highest corresponding peak area proportion A_23_ of 95.71%, further indicating that CF treatment promoted the transition of more water toward a less mobile state and resulted in a more ordered gel network with enhanced water-holding capacity. This trend is consistent with reported decreases in T_23_ of wheat starch after fermentation [56].

T_21_ reflects the state of water tightly bound to starch molecules. Both CF and LF samples showed significant decreases in A_21_, suggesting a reduction in the relative content of tightly bound water, which may have partially converted to less mobile water. T_22_ mainly exists inside the gel network or in micropores, and its state is influenced by network compactness. The CF sample had significantly lower T_22_ and higher A_22_ than the other samples (*p* < 0.05), indicating that the gel network formed by co-fermentation was more uniform and compact, capable of confining more water molecules in a less mobile form through capillary action and surface effects, thereby improving the water-holding stability of the system.

Notably, changes in T_21_ and T_22_ varied among different fermentation methods. The LF sample exhibited the longest T_21_, reflecting relatively higher mobility of its bound water, which may be related to local structural loosening induced by lactic acid bacteria fermentation. In contrast, the CF sample showed shorter relaxation times and higher proportions of confined water in both T_21_ and T_22_, indicating that co-fermentation had a more pronounced effect in promoting molecular ordering and constructing a homogeneous, dense gel network.

In summary, fermentation significantly modulated the state and distribution of water in the gel system by altering starch molecular structure and its interaction with water. Co-fermentation exhibited particularly outstanding performance in enhancing network ordering and improving water-holding capacity.

### 3.13. Digestive Property Analysis

**Figure 5:**
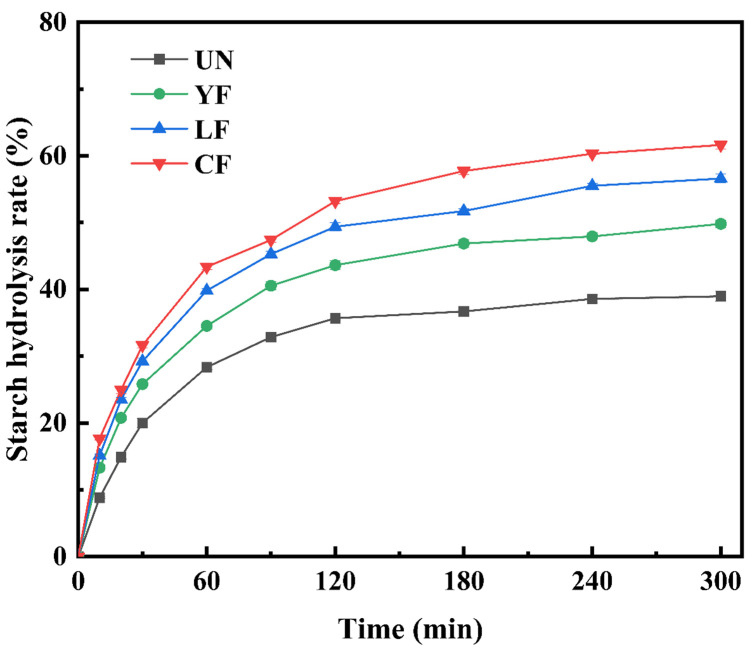
Hydrolysis Rates of millet starch extracted using different fermentation methods.

Figure 5 presents the in vitro digestibility curves of millet starch following different fermentation treatments. The UN sample exhibited the lowest hydrolysis rate throughout the digestion period, indicating a relatively stable starch structure. In contrast, all fermented samples showed progressively higher hydrolysis rates, with CF starch displaying the highest digestibility. This enhanced hydrolysis may lead to greater potential energy bioavailability, which could be significant for the development of more easily digestible cereal-based foods [57].

Table 7 presents the digestible starch contents of millet starch samples. The rapidly digestible starch (RDS) content increased significantly with fermentation (*p* < 0.05), with CF reaching the highest RDS value of 24.96%. This rise may be attributed to the disruption of crystalline barriers [58]; the decrease in crystallinity (Table 1) implies that enzymes can more easily penetrate and erode starch granules, attacking the internal α-1,4 glycosidic bonds. Meanwhile, surface erosion and pore formation—clearly visible in the SEM images (Figure 1)—provided additional entry points and increased surface area for digestive enzymes. Long chains were cleaved into shorter segments (e.g., A and B_1_ chains), which serve as ideal substrates for digestive enzymes and can be rapidly hydrolyzed. The slowly digestible starch (SDS) content also increased significantly after fermentation (*p* < 0.05). Although crystallinity decreased, the higher proportion of short amylopectin branches might have led to the formation of a short, dense gel network after gelatinization. Such a gel exhibits high viscosity, which can significantly restrict the diffusion of water and digestive enzymes, thereby physically slowing down digestion and resulting in elevated SDS levels [47]. Moreover, the resistant starch (RS) content of co-fermented starch decreased significantly (*p* < 0.05) to 46.80%. The physical barriers in RS (RS1) were partially degraded and weakened by microbial enzymes. Retrograded starch (RS3), which mainly forms through the reassociation of leached amylose molecules [59], was substantially reduced due to the lower amylose content (Table 1) and shorter molecular chains (Table 2) after fermentation, impairing the ability of amylose to retrograde and form stable double helices.

In conclusion, fermentation effectively disrupts the native structural barriers of starch—including crystalline regions, long-chain molecules, and granule integrity—transforming it from a slowly and incompletely digestible form into one that is rapidly and readily digestible. These changes directly influence the nutritional properties of fermented millet products and may offer a strategy for modulating starch digestibility in specific dietary applications, such as developing easily digestible foods for vulnerable populations or modulating postprandial glycemic responses.

## 4. Conclusions

This study investigated the effects of different fermentation treatments on the properties of millet starch. Starch was extracted using a water-washing method for comparative analysis. The results demonstrated that co-fermentation with *Lactobacillus* LP707 and yeast reduced the amylose content of millet starch. SEM observations revealed that, compared to UN, the fermented starch granules exhibited more surface imperfections such as pits, cracks, and debris, with the changes being more pronounced in the CF sample. Furthermore, while fermentation did not alter the functional groups or crystalline type of the starch, it significantly decreased the absorbance ratio and crystallinity. In addition, the CF sample showed lower gelatinization enthalpy, setback value, and final viscosity. Enhanced interactions between water molecules and the starch gel were also observed in CF, contributing to a more ordered and stable gel structure. The CF sample exhibited the highest starch hydrolysis rate, increased rapidly digestible starch content, and decreased resistant starch content, indicating improved digestibility. In conclusion, co-fermentation with *Lactobacillus* LP707 and yeast demonstrated superior effects on millet starch compared to individual fermentations, leading to improved functional properties that facilitate the fermentation process of millet-based products and enhance their applicability. Although this study investigated the effects of fermentation on millet starch using millet dough as a carrier, the processing adaptability of co-fermented starch in other food systems (such as noodles, bakery products, and beverages) was not systematically examined. In future research, we aim to apply co-fermented starch to a broader range of food systems (e.g., gluten-free bread, functional noodles, and nutritional rice flour) and comprehensively evaluate its processing adaptability, sensory quality, and storage stability.

## Figures and Tables

**Table 1:** The apparent amylose content, color parameters, particle size distribution, short-range ordered structure, and relative crystallinity of millet starch extracted using different fermentation methods.

Sample	UN	YF	LF	CF
AAC (%)	24.07 ± 0.11 ^a^	20.34 ± 0.21 ^b^	19.55 ± 0.33 ^c^	17.45 ± 0.22 ^d^
L*	95.94 ± 0.01 ^d^	96.47 ± 0.02 ^c^	97.34 ± 0.01 ^a^	97.24 ± 0.01 ^b^
a*	−0.12 ± 0.00 ^d^	−0.05 ± 0.01 ^a^	−0.08 ± 0.00 ^b^	−0.11 ± 0.01 ^c^
b*	3.90 ± 0.01 ^a^	3.19 ± 0.00 ^c^	3.54 ± 0.01 ^b^	2.61 ± 0.01 ^d^
ΔE	-	0.89 ± 0.01 ^c^	1.45 ± 0.01 ^b^	1.84 ± 0.01 ^a^
d_(0,1)_ (μm)	4.88 ± 0.04 ^b^	5.14 ± 0.03 ^a^	3.90 ± 0.02 ^c^	3.73 ± 0.07 ^d^
d_(0,5)_ (μm)	10.41 ± 0.24 ^a^	10.08 ± 0.04 ^b^	10.09 ± 0.03 ^b^	9.82 ± 0.18 ^b^
d_(0,9)_ (μm)	15.72 ± 1.41 ^a^	13.73 ± 0.16 ^b^	16.00 ± 0.12 ^a^	15.12 ± 0.55 ^ab^
SpSA (m^2^/kg)	253.47 ± 5.65 ^c^	256.27 ± 0.81 ^c^	295.43 ± 1.50 ^b^	307.80 ± 6.02 ^a^
span	1.04 ± 0.12 ^b^	0.85 ± 0.01 ^c^	1.20 ± 0.01 ^a^	1.16 ± 0.03 ^a^
R_1047/1022_	1.61 ± 0.01 ^a^	1.44 ± 0.01 ^c^	1.42 ± 0.01 ^d^	1.45 ± 0.01 ^b^
R_995/1022_	2.09 ± 0.01 ^a^	1.70 ± 0.01 ^b^	1.62 ± 0.01 ^c^	1.56 ± 0.01 ^d^
RC (%)	26.44 ± 0.10 ^a^	24.83 ± 0.08 ^b^	22.25 ± 0.09 ^c^	20.78 ± 0.12 ^d^

The values in the table are presented as mean ± standard deviation (*n* = 3). Values in the same row with different letters are significantly different (*p* < 0.05). AAC: apparent amylose content; L*: lightness; a*: red/green value; b*: yellow/blue value; ΔE: color difference; d_(0.1)_: particle diameter at 10% cumulative volume; d_(0.5)_: median particle diameter; d_(0.9)_: particle diameter at 90% cumulative volume; SpSA: specific surface area; span: span value; R_1047_/_1022_: absorbance ratio at 1047 cm^−1^ to 1022 cm^−1^; R_995_/_1022_: absorbance ratio at 995 cm^−1^ to 1022 cm^−1^; RC: relative crystallinity. UN: unfermented millet starch; YF: millet starch fermented with *Lactobacillus* LP707; LF: millet starch fermented with yeast; CF: millet starch co-fermented with *Lactobacillus* LP707 and yeast.

**Table 2:** The chain length distribution, molecular weight, and gyration radius of millet starch extracted using different fermentation methods.

Sample	UN	YF	LF	CF
Chain length distribution				
A (DP6–12)	24.02 ± 0.14 ^d^	24.43 ± 0.10 ^c^	24.86 ± 0.15 ^b^	25.39 ± 0.13 ^a^
B_1_ (DP13–24)	50.36 ± 0.04 ^d^	50.55 ± 0.12 ^c^	50.75 ± 0.09 ^b^	51.02 ± 0.12 ^a^
B_2_ (DP25–36)	13.09 ± 0.06 ^a^	12.98 ± 0.04 ^a^	12.65 ± 0.12 ^b^	12.33 ± 0.04 ^c^
B_3_ (DP ≥ 37)	12.53 ± 0.10 ^a^	12.04 ± 0.03 ^b^	11.74 ± 0.06 ^c^	11.26 ± 0.03 ^d^
Molecular weight and gyration radius				
Mw (10^4^ g/mol)	12.36 ± 0.31 ^a^	11.80 ± 0.33 ^b^	7.70 ± 0.14 ^c^	5.86 ± 0.02 ^d^
Mn (10^4^ g/mol)	3.31 ± 0.10 ^b^	3.46 ± 0.03 ^a^	1.73 ± 0.02 ^c^	1.34 ± 0.02 ^d^
(Mw/Mn)	3.74 ± 0.02 ^b^	3.41 ± 0.07 ^c^	4.44 ± 0.03 ^a^	4.36 ± 0.05 ^a^
Rz (nm)	211.38 ± 1.88 ^a^	205.39 ± 1.59 ^b^	178.38 ± 1.74 ^c^	161.75 ± 0.94 ^d^

The values in the table are presented as mean ± standard deviation (*n* = 3). Values in the same row with different letters are significantly different (*p* < 0.05). A: A-chains (degree of polymerization 6–12); B_1_: B_1_-chains (degree of polymerization 13–24); B_2_: B_2_-chains (degree of polymerization 25–36); B_3_: B_3_-chains (degree of polymerization ≥37); Mw: weight-average molecular weight; Mn: number-average molecular weight; Mw/Mn: polydispersity index; Rz: radius of gyration. UN: unfermented millet starch; YF: millet starch fermented with *Lactobacillus* LP707; LF: millet starch fermented with yeast; CF: millet starch co-fermented with *Lactobacillus* LP707 and yeast.

**Table 3:** Thermal characteristic parameters of millet starch extracted using different fermentation methods.

Sample	UN	YF	LF	CF
To (°C)	68.84 ± 0.38 ^a^	67.02 ± 0.93 ^b^	66.77 ± 0.81 ^b^	64.51 ± 0.36 ^c^
Tp (°C)	75.89 ± 0.65 ^b^	75.94 ± 0.27 ^b^	79.01 ± 0.99 ^a^	77.46 ± 1.22 ^ab^
Tc (°C)	86.07 ± 0.58 ^b^	88.09 ± 0.84 ^ab^	89.94 ± 1.32 ^a^	88.99 ± 1.58 ^a^
ΔH (J/g)	10.91 ± 0.92 ^a^	9.02 ± 0.91 ^b^	8.80 ± 0.38 ^b^	7.32 ± 0.48 ^c^

The values in the table are presented as mean ± standard deviation (*n* = 3). Values in the same row with different letters are significantly different (*p* < 0.05). To: onset temperature; Tp: peak temperature; Tc: conclusion temperature; ΔH: gelatinization enthalpy. UN: unfermented millet starch; YF: millet starch fermented with *Lactobacillus* LP707; LF: millet starch fermented with yeast; CF: millet starch co-fermented with *Lactobacillus* LP707 and yeast.

**Table 4:** Gelatinization characteristics of millet starch extracted using different fermentation methods.

Sample	UN	YF	LF	CF
PT (°C)	77.25 ± 0.52 ^c^	77.78 ± 0.45 ^c^	81.60 ± 0.05 ^a^	79.88 ± 0.80 ^b^
PV (cP)	3629.00 ± 25.51 ^a^	3541.00 ± 31.19 ^b^	2567.00 ± 23.58 ^d^	2679.33 ± 26.27 ^c^
TV (cP)	1836.67 ± 15.18 ^b^	1910.00 ± 32.14 ^a^	1501.33 ± 14.22 ^c^	1320.00 ± 36.17 ^d^
FV (cP)	3616.00 ± 28.62 ^a^	3634.33 ± 29.14 ^a^	2920.00 ± 20.07 ^b^	2544.33 ± 40.28 ^c^
BD (cP)	1792.33 ± 14.19 ^a^	1631.00 ± 10.58 ^b^	1065.67 ± 26.08 ^d^	1359.33 ± 15.95 ^c^
SB (cP)	1779.33 ± 14.98 ^a^	1724.33 ± 15.50 ^b^	1418.67 ± 13.20 ^c^	1224.33 ± 24.83 ^d^

The values in the table are presented as mean ± standard deviation (*n* = 3). Values in the same row with different letters are significantly different (*p* < 0.05). PT: Pasting Temperature; PV: Peak Viscosity; TV: Trough Viscosity; FV: Final Viscosity; BD: Breakdown; SB: Setback. UN: unfermented millet starch; YF: millet starch fermented with *Lactobacillus* LP707; LF: millet starch fermented with yeast; CF: millet starch co-fermented with *Lactobacillus* LP707 and yeast.

**Table 5:** Gel texture characteristics of millet starch extracted using different fermentation methods.

Sample	UN	YF	LF	CF
Hardness (gf)	49.69 ± 0.68 ^a^	49.55 ± 0.29 ^a^	49.23 ± 0.2 ^a^	49.22 ± 0.11 ^a^
Springiness (%)	0.86 ± 0.02 ^a^	0.78 ± 0.01 ^b^	0.70 ± 0.01 ^c^	0.77 ± 0.02 ^b^
Chewiness (gf)	40.75 ± 1.54 ^a^	36.55 ± 0.78 ^b^	31.45 ± 0.29 ^c^	34.58 ± 1.37 ^b^
Gumminess (gf)	47.23 ± 0.84 ^a^	46.83 ± 0.72 ^a^	45.06 ± 0.67 ^b^	44.89 ± 0.39 ^b^
Cohesiveness (%)	0.95 ± 0.01 ^a^	0.95 ± 0.02 ^a^	0.92 ± 0.01 ^b^	0.91 ± 0.01 ^b^
Resilience (%)	0.6 ± 0.27 ^a^	0.56 ± 0.21 ^a^	0.56 ± 0.05 ^a^	0.68 ± 0.02 ^a^

The values in the table are presented as mean ± standard deviation (*n* = 3). Values in the same row with different letters are significantly different (*p* < 0.05). UN: unfermented millet starch; YF: millet starch fermented with *Lactobacillus* LP707; LF: millet starch fermented with yeast; CF: millet starch co-fermented with *Lactobacillus* LP707 and yeast.

**Table 6:** Moisture distribution of millet starch extracted using different fermentation methods.

Sample	UN	YF	LF	CF
T_21_ (ms)	1.01 ± 0.15 ^b^	1.09 ± 0.08 ^b^	1.78 ± 0.16 ^a^	1.12 ± 0.15 ^b^
T_22_ (ms)	9.37 ± 0.27 ^b^	10.77 ± 0.16 ^a^	10.69 ± 0.16 ^a^	7.05 ± 0.23 ^c^
T_23_ (ms)	231.01 ± 0.00 ^a^	200.92 ± 0.00 ^b^	132.19 ± 0.00 ^c^	132.19 ± 0.00 ^c^
A_21_ (%)	4.67 ± 0.21 ^a^	2.64 ± 0.18 ^b^	2.12 ± 0.07 ^c^	1.32 ± 0.23 ^d^
A_22_ (%)	2.55 ± 0.06 ^d^	2.71 ± 0.07 ^c^	2.84 ± 0.06 ^b^	2.97 ± 0.05 ^a^
A_23_ (%)	92.79 ± 0.27 ^d^	94.65 ± 0.11 ^c^	95.04 ± 0.12 ^b^	95.71 ± 0.18 ^a^

The values in the table are presented as mean ± standard deviation (*n* = 3). Values in the same row with different letters are significantly different (*p* < 0.05). T_21_: Bound water (approximately 0.1–10 ms); T_22_: weakly bound water (approximately 10–100 ms); T_23_: free water (generally >100 ms); A_21_: Peak area corresponding to T_21_; A_22_: Peak area corresponding to T_22_; A_23_: Peak area corresponding to T_23_. UN: unfermented millet starch; YF: millet starch fermented with *Lactobacillus* LP707; LF: millet starch fermented with yeast; CF: millet starch co-fermented with *Lactobacillus* LP707 and yeast.

**Table 7:** Digestive properties of millet starch extracted using different fermentation methods.

Sample	UN	YF	LF	CF
RDS (%)	14.89 ± 0.31 ^d^	20.75 ± 0.33 ^c^	23.51 ± 0.26 ^b^	24.96 ± 0.55 ^a^
SDS (%)	20.79 ± 0.25 ^d^	22.87 ± 0.93 ^c^	25.86 ± 0.41 ^b^	28.24 ± 0.85 ^a^
RS (%)	64.32 ± 0.33 ^a^	56.38 ± 0.65 ^b^	50.62 ± 0.59 ^c^	46.80 ± 0.34 ^d^

The values in the table are presented as mean ± standard deviation (*n* = 3). Values in the same row with different letters are significantly different (*p* < 0.05). RDS: rapidly digestible starch; SDS: slowly digestible starch; RS: resistant starch. UN: unfermented millet starch; YF: millet starch fermented with *Lactobacillus* LP707; LF: millet starch fermented with yeast; CF: millet starch co-fermented with *Lactobacillus* LP707 and yeast.

## Data Availability

The original contributions presented in this study are included in the article/Supplementary Materials. Further inquiries can be directed to the corresponding author.

## Supplementary Materials

The following supporting information can be downloaded at https://www.mdpi.com/article/10.3390/foods15071186/s1: Figure S1. Effects of different fermentation methods on the hydrolysis rate of millet steamed bread. Table S1. Effects of different fermentation methods on the textural properties of millet steamed bread. Table S2. Effects of different fermentation methods on the digestive properties of millet steamed bread.
