# Supplementary material for: Effects of Co-Fermentation with Lactobacillus and Yeast on the Structural and Physicochemical Properties of Millet Starch
Source: Foods. 2026 Apr 1;15(7):1186. doi: 10.3390/foods15071186 (PMC13074191; doi:10.3390/foods15071186)
Supplement: Supplementary file 1 [file foods-15-01186-s001.zip › foods-4193215-supplementary.pdf]

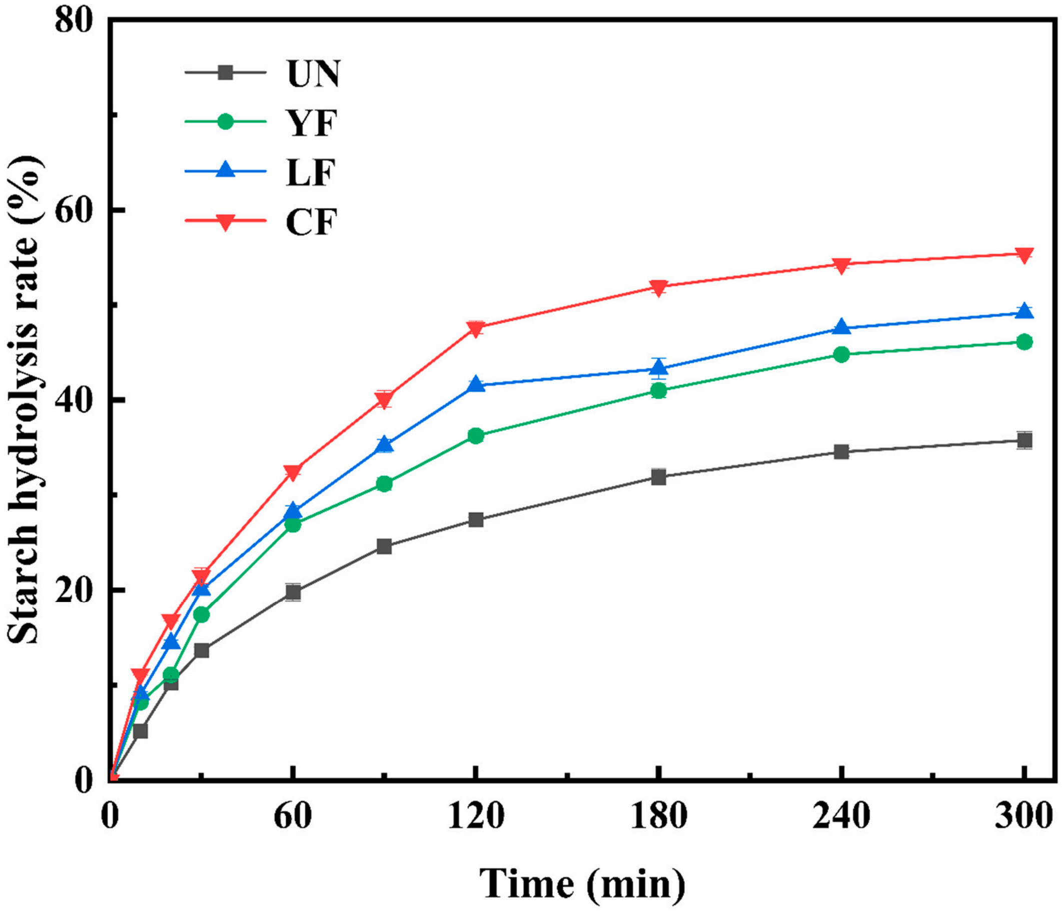

Fig. S1. Effects of different fermentation methods on the hydrolysis rate of millet steamed bread.
UN: naturally fermented millet steamed bread;
YF: millet steamed bread fermented with *Lactobacillus* LP707;
LF: millet steamed bread fermented with yeast;
CF: millet steamed bread co-fermented with *Lactobacillus* LP707 and yeast.

Table S1. Effects of different fermentation methods on the textural properties of millet steamed bread.

Sample	UN	YF	LF	CF
Hardness (gf)	243.96±8.71^{a}	94.00±4.93^{c}	109.24±8.60^{b}	77.85±4.02^{d}
Springiness (%)	0.71±0.03^{c}	0.74±0.03^{bc}	0.80±0.02^{ab}	0.84±0.04^{a}
Chewiness (gf)	84.66±1.05^{a}	45.83±0.51^{c}	62.45±3.45^{b}	42.85±2.47^{c}
Gumminess (gf)	120.85±7.96^{a}	64.16±2.72^{c}	84.03±4.72^{b}	28.74±4.29^{d}
Cohesiveness (%)	0.49±0.04^{b}	0.66±0.04^{a}	0.72±0.04^{a}	0.66±0.03^{a}
Resilience (%)	0.30±0.03^{c}	0.32±0.02^{bc}	0.39±0.04^{b}	0.48±0.05^{a}

The values in the table are presented as mean ± standard deviation (n = 3). Different lowercase letters denote statistically significant differences between groups ($p < 0.05$), whereas identical lowercase letters indicate no statistically significant differences.
UN: naturally fermented millet steamed bread;
YF: millet steamed bread fermented with *Lactobacillus* LP707;

LF: millet steamed bread fermented with yeast;
CF: millet steamed bread co-fermented with *Lactobacillus* LP707 and yeast.

Table S2. Effects of different fermentation methods on the digestive properties of millet steamed bread.

Sample	UN	YF	LF	CF
RDS (%)	10.24±0.30^{d}	11.07±0.21^{c}	14.41±0.31^{b}	16.84±0.14^{a}
SDS (%)	17.15±0.27^{d}	25.16±0.43^{c}	27.11±0.54^{b}	30.79±0.66^{a}
RS (%)	72.61±0.28^{a}	61.76±0.54^{b}	58.81±0.62^{c}	52.37±0.67^{d}

The values in the table are presented as mean ± standard deviation (n = 3). Different lowercase letters denote statistically significant differences between groups (p < 0.05), whereas identical lowercase letters indicate no statistically significant differences.
UN: naturally fermented millet steamed bread;
YF: millet steamed bread fermented with *Lactobacillus* LP707;
LF: millet steamed bread fermented with yeast;
CF: millet steamed bread co-fermented with *Lactobacillus* LP707 and yeast.
